# Analysis of the correlation between Hashimoto’s thyroiditis and food intolerance

**DOI:** 10.3389/fnut.2024.1452371

**Published:** 2024-09-30

**Authors:** Manli Yan, Hai Wu, Kaiyuan Zhang, Ping Gong, Yiting Wang, Hua Wei

**Affiliations:** ^1^Second Clinical Medical College, Guangzhou University of Chinese Medicine, Guangzhou, China; ^2^Meizhou Maternity and Child HealthCare Hospital, Meizhou, China; ^3^Department of Endocrinology, Guangdong Provincial Hospital of Chinese Medicine, Guangdong, China; ^4^State Key Laboratory of Dampness Syndrome of Chinese Medicine, The Second Affiliated Hospital of Guangzhou University of Chinese Medicine, Guangzhou, China

**Keywords:** Hashimoto’s thyroiditis, food intolerance, food-specific IgG antibodies, thyroid peroxidase antibodies, thyroglobulin antibodies

## Abstract

**Objective:**

This study aims to explore the correlation between patients with Hashimoto’s thyroiditis and food intolerance.

**Methods:**

A total of 172 subjects who visited Guangdong Provincial Hospital of Traditional Chinese Medicine between January 2020 and March 2023 were selected and tested for 90 food-specific IgG antibodies. The study group composed 85 individuals diagnosed with Hashimoto’s thyroiditis, while the control group consisted of 87 healthy individuals. Data were analyzed to determine the correlation between Hashimoto’s thyroiditis and food intolerance.

**Results:**

Among the 85 patients with Hashimoto’s thyroiditis, 97.65% exhibited food intolerance, with an average of 15.76 ± 10.61 types of food intolerances. The most common intolerances were to eggs (75.29%), bok choy (71.76%), and milk (65.88%), each exceeding a 60% intolerance rate. In the control group of 87 healthy individuals, the intolerance rate was 95.40%, with an average of 9.57 ± 8.90 types of food intolerances. The most prevalent intolerances in the control group were to bok choy (54.02%) and eggs (52.87%), each exceeding a 50% intolerance rate.

**Conclusion:**

The findings suggest that patients with Hashimoto’s thyroiditis are more likely to develop food intolerance compared to the healthy population, which may indicate a correlation between Hashimoto’s thyroiditis and food intolerance. Different dietary patterns may affect the activity of the thyroid axis and may even be the cause of autoimmune thyroid disease. The technique of detecting food intolerance IgG antibodies has the potential to be an important reference for dietary interventions in patients with Hashimoto’s thyroiditis.

## 1 Introduction

Hashimoto’s thyroiditis (HT) (1), also known as chronic lymphocytic thyroiditis, is a common autoimmune disorder characterized by widespread thyroid enlargement and the presence of high levels of thyroid peroxidase antibodies (TPOAb) and thyroglobulin antibodies (TgAb) in the serum. As the disease progresses, the thyroid gland is gradually destroyed, leading to abnormal gland function, with about 20–30% (2) of patients developing hypothyroidism. Certain patients (3) display extremely high antibody titers, significant changes in thyroid texture, and persistent thyroid function abnormalities, including pronounced fluctuations in TSH levels (fluctuating between hyperthyroidism and hypothyroidism). These cases present significant clinical challenges, as conventional treatments often fail to maintain normal thyroid function and reduce thyroid-associated antibodies, highlighting the urgent need for innovative diagnostic and therapeutic approaches.

It has been found that (4, 5) the immune response is more pronounced in patients with Hashimoto’s thyroiditis compared to the normal population and is closely related to the gut flora (6). Due to the strong link between diet and gut flora, we hope to find effective interventions and treatments by modifying diet. Food intolerance (FI) (7) is an IgG-mediated immune response that can cause inflammation in multiple tissues. If dietary structures are not promptly adjusted to mitigate this immune response, it can lead to abnormal organ function. An et al. (8) conducted food-specific IgG antibody testing on 153 HT patients and found a positive rate of 64.05%. They concluded that food intolerance is common in HT patients. The aim of this study was to investigate the relationship between Hashimoto’s thyroiditis and food intolerance (specific IgG antibody test for 90 foods).

## 2 Materials and methods

### 2.1 Subjects

A total of 172 subjects who visited Guangdong Provincial Hospital of Traditional Chinese Medicine between January 2020 and March 2023 were selected and tested for 90 food-specific IgG antibodies. Among them, 85 were diagnosed with Hashimoto’s thyroiditis (HT group), and 87 were included in a healthy control group (Non-HT group).

#### 2.1.1 Diagnostic criteria for Hashimoto’s thyroiditis

In conjunction with the 2008 Chinese Guidelines for the Diagnosis and Treatment of Thyroid Diseases-Thyroiditis (9) and the American Thyroid Association’s (10) manual on Hashimoto’s thyroiditis: Any patient with diffuse thyroid enlargement, a tougher gland texture, especially with an enlarged pyramidal lobe, should be suspected of HT. Diagnosis can be confirmed if serum TPOAb and TgAb are positive. Fine Needle Aspiration Cytology (FNAC) has diagnostic value. Clinical or subclinical hypothyroidism further supports the diagnosis.

In this study, the diagnosis of HT was mainly made by thyroid ultrasound presenting changes characteristic of HT (e.g., abundant blood flow signal, diffuse enlargement, etc.) and positive TPOAb and TgAb.

#### 2.1.2 Criteria for assessing the healthy population

The healthy control group (Non-HT group) was defined as people whose thyroid-related antibody (TPOAb and TgAb) tests were suggestive of negativity and whose thyroid ultrasound did not show Hashimoto’s thyroiditis-specific changes. In addition, in order to minimize the interference of other autoimmune diseases with the results of this study, we excluded subjects with a clear diagnosis of other autoimmune diseases, such as type 1 diabetes mellitus, Addison’s syndrome, and systemic lupus erythematosus. Sensitized subjects with multiple allergies were also excluded (mainly to multiple IgE antibodies).

### 2.2 Methods

#### 2.2.1 Collection of clinical data

We meticulously recorded data concerning the subjects’ age, gender, thyroid ultrasound findings, thyroid function test results, and levels of thyroid-related antibodies (TgAb, TPOAb).

#### 2.2.2 Measurement of thyroid-related indicators

Blood samples were collected from all subjects, and quality control was conducted by the Laboratory Department of Guangdong Provincial Hospital of Traditional Chinese Medicine. Free triiodothyronine (FT3), free thyroxine (FT4), and thyroid-stimulating hormone (TSH) levels were measured using chemiluminescence, while TgAb and TPOAb levels were measured using electrochemiluminescence.

The normal reference ranges are as follows: FT3: 3.5–6.5 pmol/L, FT4: 11.5–22.7 pmol/L, TSH: 0.51–4.97 mIU/L, TgAb: < 60 U/mL, TPOAb: < 60 U/mL.

#### 2.2.3 Measurement of food-specific IgG antibodies

Blood samples were collected from all subjects after fasting for 12 h and were sent to the Guangzhou JingYu Center for Clinical Laboratory. Food-specific IgG antibody test kits (HOB Biotech Group) were used to measure serum concentrations of 90 food-specific antibodies and to perform grading.

The grading standards were as follows: < 50 U/mL as negative; ≥ 50 U/mL as positive; under the positive result, the antibody titer level was graded as follows: 50–100 U/mL as level I intolerance; 100–200 U/mL as level II intolerance; ≥ 200 U/mL as level III intolerance.

The 90 food items total include: Cheddar cheese, White soft cheese, Beef, Chicken, Pork, Eggs, Lamb, Turkey, Yogurt, Chocolate, Corn, Rice, Wheat, Soybeans, Lemons, Spinach, Green beans, Millet, Almonds, Sesame seeds, Cashews, Peanuts, Dried, celery, Eggplants, Green peppers, Parsley, Cabbage, Carrots, Cauliflower, Young peas, Sunflower seeds, Black walnuts, Blueberries, Pineapple, Oranges, Peaches, Apples, Durian, Mango, Banana, Grape Grapefruit, Strawberry, Cantaloupe, Malt, Rye, Yeast, Cane sugar, Butter, Cinnamon, Mustard, Honey, Coffee, Oats, Barley, Buckwheat, Broccoli, Mixed colored peas, Garlic, Onion, Cola beans, Black tea, Milk, Crab, Shrimp, Cod, Goat’s milk, Clam, Salmon, Sardines, Cutlassfish, Scallop, Lobster, Grass carp, Oysters, Trout, Tuna, Red pepper garlic, Spring onion, Lettuce, Cucumber, Potato, Pumpkin, Sweet potato, Cilantro, Bok choy, Tomato, Mushroom, Watermelon, and Olive.

#### 2.2.4 Statistical

Data were analyzed using IBM Statistical Package for the Social Sciences, Version 26.0. The level of significance for tests was set at α = 0.05. Count data were expressed as frequencies and percentages. Comparisons between groups were made using the Chi-square test or Fisher’s exact test, with *P* < 0.05 indicating statistical significance.

## 3 Results

### 3.1 General characteristics of patients with Hashimoto’s thyroiditis and healthy individuals

Among the 85 patients with Hashimoto’s thyroiditis, TgAb levels averaged (391.42 ± 528.82) U/mL, and TPOAb levels averaged (864.41 ± 518.45) U/mL. The average age was (35.94 ± 14.10) years, with 73 females and 12 males. In the Non-HT group of 87 individuals, the average age was (44.39 ± 13.47) years, with 63 females and 24 males.

### 3.2 Comparison of intolerance to 90 food groups

Among the 90 food items tested, the intolerance rates for cheddar cheese, white cottage cheese, eggs, yogurt, wheat, carrots, pineapple, bananas, cantaloupe, malt, butter, cinnamon, mustard, cow’s milk, crab, goat’s milk, scallops, grass carp, cucumber, pumpkin, and mushrooms showed significant differences between the two groups (see Table 1). Notably, none of the subjects in this study exhibited intolerance to turkey meat, strawberries, cola beans, or sweet potato (see Table 1).

**TABLE 1:** Food intolerance profile of 172 subjects for 90 food-specific IgG antibody tests.

Items	HT group (*n* = 85)				Non-HT group (*n* = 87)				χ ^2^	*P* *
	Level 0	Level I	Level II	Level III	Level 0	Level I	Level II	Level III		
Cheddar cheese	55	10	11	9	75	7	5	0	15.595	0.001
White soft cheese	43	14	15	13	67	12	5	3	16.619	0.001
Beef	81	4	0	0	83	4	0	0	0.001	1
Chicken	83	2	0	0	85	2	0	0	0.000	1
Pork	84	1	0	0	86	1	0	0	0.000	1
Eggs	21	26	21	17	41	27	10	9	12.814	0.005
Lamb	83	2	0	0	86	1	0	0	0.000	0.984
Turkey	85	0	0	0	87	0	0	0		
Yogurt	47	15	17	6	71	12	3	1	18.808	0.000
Chocolate	68	14	2	1	75	8	3	1	2.430	0.500
Corn	76	9	0	0	82	4	1	0	2.999	0.159
Rice	65	16	4	0	71	13	3	0	0.754	0.709
Wheat	66	9	7	3	78	8	1	0	8.066	0.030
Soybeans	68	14	3	0	77	8	1	1	4.008	0.231
Lemons	77	7	1	0	81	5	1	0	0.670	0.780
Spinach	83	2	0	0	86	1	0	0	0.000	0.984
Green beans	74	10	1	0	74	12	1	0	0.416	0.910
Millet	70	14	1	0	67	18	2	0	0.956	0.620
Almonds	43	30	9	3	61	20	4	2	7.189	0.059
Sesame seeds	84	1	0	0	84	1	2	0	1.847	0.747
Cashews	57	23	5	0	66	18	3	0	1.749	0.461
Peanuts	46	21	14	4	61	15	9	2	4.789	0.178
Dried celery	84	1	0	0	84	1	2	0	1.847	0.747
Eggplants	84	1	0	0	85	2	0	0	0.000	1.000
Green peppers	83	1	1	0	83	4	0	0	2.564	0.368
Parsley	72	13	0	0	79	7	1	0	2.997	0.159
Cabbage	84	1	0	0	83	3	1	0	1.853	0.621
Carrots	72	10	3	0	81	2	4	0	6.019	0.048
Cauliflower	85	0	0	0	86	1	0	0	0.000	1.000
Young peas	78	6	1	0	84	2	1	0	2.335	0.272
Sunflower seeds	60	17	6	2	68	12	6	1	1.782	0.640
Black walnuts	79	6	0	0	84	3	0	0	0.519	0.471
Blueberries	84	1	0	0	87	0	0	0	0.000	0.991
Pineapple	60	21	3	1	75	11	1	0	6.598	0.048
Oranges	78	6	1	0	80	7	0	0	1.047	0.889
Peaches	76	9	0	0	84	3	0	0	3.377	0.078
Apples	84	0	1	0	85	2	0	0	2.546	0.497
Durian	84	1	0	0	83	4	0	0	0.777	0.378
Mango	85	0	0	0	86	1	0	0	0.000	1.000
Banana	57	9	10	9	79	3	4	1	15.442	0.001
Grape	84	0	1	0	85	1	1	0	1.159	1.000
Grapefruit	76	9	0	0	80	7	0	0	0.329	0.609
Strawberry	85	0	0	0	87	0	0	0		
Cantaloupe	59	22	4	0	75	11	1	0	7.168	0.022
Malt	47	28	9	1	63	21	3	0	7.133	0.044
Rye	71	10	2	2	80	7	0	0	4.298	0.155
Yeast	82	2	1	0	85	2	0	0	1.089	0.808
Cane sugar	80	4	1	0	85	2	0	0	1.721	0.323
Butter	57	16	11	1	77	8	2	0	12.803	0.002
Cinnamon	45	39	1	0	62	25	0	0	6.647	0.018
Mustard	52	25	7	1	73	12	2	0	11.641	0.004
Honey	73	11	1	0	77	8	2	0	0.973	0.603
Coffee	85	0	0	0	84	2	1	0	2.546	0.497
Oats	76	9	0	0	80	7	0	0	0.329	0.609
Barley	76	6	3	0	84	2	1	0	3.205	0.201
Buckwheat	82	3	0	0	82	3	2	0	1.688	0.603
Broccoli	65	16	4	0	74	8	5	0	3.337	0.183
Mixed colored peas	71	13	1	0	80	6	1	0	3.279	0.138
Garlic	66	14	5	0	74	10	3	0	1.604	0.496
Onion	59	20	6	0	70	11	6	0	3.528	0.178
Cola beans	85	0	0	0	87	0	0	0		
Black tea	85	0	0	0	86	1	0	0	0.000	1.000
Milk	29	14	17	25	56	13	9	9	18.584	0.000
Crab	57	26	2	0	72	11	3	1	9.010	0.012
Shrimp	72	12	1	0	78	8	1	0	1.249	0.673
Cod	71	11	3	0	75	9	3	0	0.385	0.885
Goat’s milk	47	14	14	10	70	13	3	1	19.019	0.000
Clam	64	21	0	0	72	15	0	0	1.448	0.263
Salmon	81	4	0	0	85	2	0	0	0.198	0.657
Sardines	74	8	2	1	80	6	1	0	1.859	0.583
Cutlassfish	79	5	1	0	87	0	0	0	6.166	0.013
Scallop	83	2	0	0	87	0	0	0	0.530	0.467
Lobster	78	7	0	0	83	4	0	0	0.950	0.368
Grass carp	68	14	3	0	80	7	0	0	5.842	0.034
Oysters	71	12	2	0	77	7	2	1	2.566	0.407
Trout	83	2	0	0	87	0	0	0	0.530	0.467
Tuna	75	9	1	0	79	6	2	0	1.091	0.569
Red pepper garlic	74	11	0	0	82	5	0	0	2.637	0.121
Spring onion	73	12	0	0	76	10	1	0	1.169	0.653
Lettuce	84	1	0	0	83	4	0	0	0.777	0.378
Cucumber	75	10	0	0	84	3	0	0	4.256	0.046
Potato	50	29	6	0	67	15	5	0	6.993	0.031
Pumpkin	38	36	10	1	63	16	7	1	14.688	0.001
Sweet potato	85	0	0	0	87	0	0	0		
Cilantro	69	14	2	0	75	10	2	0	1.013	0.722
Bok choy	24	50	10	1	40	40	7	0	6.534	0.061
Tomato	74	9	2	0	75	10	2	0	0.184	1.000
Mushroom	65	17	3	0	80	7	0	0	8.263	0.008
Watermelon	75	10	0	0	81	6	0	0	1.208	0.304
Olive	83	2	0	0	86	1	0	0	0.000	0.984

Values are frequency counts. *P* < 0.05 was deemed significant.

*Comparisons between groups were made using the Chi-square test.

### 3.3 Distribution of food intolerance in serum of HT patients

A total of 85 patients with HT were included in the study, 83 of whom exhibited elevated food-specific IgG antibodies, resulting in a positive detection rate of 97.65%. The average number of intolerances per patient was (15.76 ± 10.61), with a range from 0 to 57 types. Among the 90 food items tested, the top fourteen with the highest positive rates were: eggs (75.29%), bok choy (71.76%), milk (65.88%), pumpkin (55.29%), white soft cheese (49.41%), almonds (49.41%), cinnamon (47.06%), peanuts (45.88%), yogurt (44.71%), malt (44.71%), goat’s milk (44.71%), potatoes (44.71%), mustard (38.82%), and cheddar cheese (35.29%). The highest rates of intolerance were primarily to eggs and dairy products (including cow’s milk, yogurt, goat’s milk, soft white cheese, and cheddar cheese).

**FIGURE 1:**
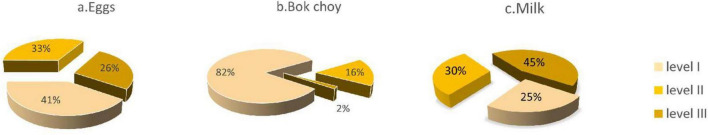
Proportion of severity levels for the top three foods with the highest positive intolerance rates in the HT group. **(a)** For eggs: 26 patients (41%) exhibited level I intolerance, 21 patients (33%) exhibited level II intolerance, and 17 patients (26%) exhibited level III intolerance. **(b)** For bok choy: 50 patients (82%) exhibited level I intolerance, 10 patients (16%) exhibited level II intolerance, and 1 patient (2%) exhibited level III intolerance. **(c)** For milk: 14 patients (25%) exhibited level I intolerance, 17 patients (30%) exhibited level II intolerance, and 25 patients (45%) exhibited level III intolerance.

Analysis of the severity levels of intolerance for the three foods with the highest positive rates revealed the following: For eggs, 26 patients (41%) exhibited level I intolerance, 21 patients (33%) exhibited level II intolerance, and 17 patients (26%) exhibited level III intolerance. For bok choy, 50 patients (82%) exhibited level I intolerance, 10 patients (16%) exhibited level II intolerance, and 1 patient (2%) exhibited level III intolerance. For cow’s milk, 14 patients (25%) exhibited level I intolerance, 17 patients (30%) exhibited level II intolerance, and 25 patients (45%) exhibited level III intolerance (see Figure 1).

#### 3.3.1 Distribution of the top 14 foods with the highest positive rates among Hashimoto’s thyroiditis patients by gender

An analysis comparing the food intolerance rates of the top 14 items among different genders within the HT group revealed that only potatoes showed significant differences between genders (see Table 2).

**TABLE 2:** Food intolerance among different genders in the HT group.

Items	Male		Female		*P* *
	Negatives	Positive	Negatives	Positive	
Eggs	2	10	19	54	0.722
Bok choy	1	11	23	50	0.165
Milk	4	8	25	48	1.000
Pumpkin	3	9	35	38	0.138
White soft cheese	6	6	37	36	0.965
Almonds	5	7	38	35	0.505
Cinnamon	5	7	40	33	0.398
Peanuts	4	8	42	31	0.119
Yogurt	6	6	41	32	0.691
Malt	5	7	42	31	0.306
Goat’s milk	5	7	42	31	0.306
Potato	1	11	49	24	< 0.001
Mustard	6	6	46	27	0.525
Cheddar cheese	6	6	49	24	0.330

Values are frequency counts. *P* < 0.05 was deemed significant.

*Comparisons between groups were made using the Fisher’s exact test.

#### 3.3.2 Distribution of the top 14 foods with the highest positive rates among Hashimoto’s thyroiditis patients by age

The analysis of food intolerance rates for the top 14 items among different age groups within the HT group revealed significant variations in the positive rates for eggs, cow’s milk, soft white cheese, yogurt, goat’s milk, and cheddar cheese across different age brackets (see Table 3).

**TABLE 3:** Food intolerance among different age in the HT group.

Items	Age ≤ 30 years		Age 30–50 years		Age > 50 years		χ ^2^	*P* *
	Negatives	Positive	Negatives	Positive	Negatives	Positive		
Eggs	2	33	8	26	11	5	21.743	< 0.001
Bok choy	10	25	8	26	6	10	1.129	0.572
Milk	3	32	16	18	10	6	18.429	< 0.001
Pumpkin	13	22	19	15	6	10	2.864	0.239
White soft cheese	8	27	22	12	13	3	19.496	< 0.001
Almonds	16	19	19	15	8	8	0.716	0.699
Cinnamon	19	16	20	14	6	10	2.029	0.363
Peanuts	18	17	18	16	10	6	0.574	0.751
Yogurt	12	23	21	13	14	2	13.538	< 0.001
Malt	17	18	23	11	7	9	3.601	0.165
Goat’s milk	11	24	23	11	13	3	14.524	< 0.001
Potato	21	14	19	15	10	6	0.231	0.891
Mustard	21	14	24	10	7	9	3.334	0.189
Cheddar cheese	15	20	24	10	16	0	16.558	< 0.001

Values are frequency counts. *P* < 0.05 was deemed significant.

*Comparisons between groups were made using the Chi-square test.

### 3.4 Distribution of food intolerance in serum of the healthy population

This study included 87 healthy individuals, 83 of whom showed elevated levels of food-specific IgG antibodies, resulting in a positive detection rate of 95.40%. The average number of food intolerances per individual was (9.57 ± 8.90), with a range from none to as many as 41 different types. Among the 90 foods tested, the fourteen with the highest positive rates were bok choy (54.02%), eggs (52.87%), milk (35.63%), almonds (29.89%), peanuts (29.89%), cinnamon (28.74%), malt (27.59%), pumpkin (27.59%), cashews (24.14%), soft white cheese (22.99%), millet (22.99%), potatoes (22.99%), sunflower seeds (21.84%), and onions (19.54%).

**FIGURE 2:**
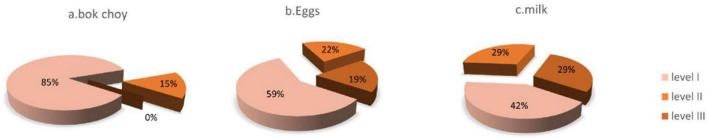
Proportion of severity levels for the top three foods with the highest positive intolerance rates in the non-HT group. **(a)** For bok choy: 40 individuals (85%) exhibited level I intolerance, 7 individuals (15%) exhibited level II intolerance, and none (0%) exhibited level III intolerance. **(b)** For eggs: 27 individuals(59%) exhibited level I intolerance, 10 individuals (22%) exhibited level II intolerance, and 9 individuals (19%) exhibited level III intolerance. **(c)** For milk: 13 individuals (42%) exhibited level I intolerance, 9 individuals (29%) exhibited level II intolerance, and 9 individuals (29%) exhibited level III intolerance.

Analyzing the three foods with the highest rates of food intolerance: For bok choy, 40 individuals (85%) exhibited level I intolerance, 7 individuals (15%) exhibited level II intolerance, and none (0%) exhibited level III intolerance. For eggs, 27 individuals (59%) exhibited level I intolerance, 10 individuals (22%) exhibited level II intolerance, and 9 individuals (19%) exhibited level III intolerance. For cow’s milk, 13 individuals (42%) exhibited level I intolerance, 9 individuals (29%) exhibited level II intolerance, and 9 individuals (29%) exhibited level III intolerance (see Figure 2).

## 4 Discussion

Xue (11) and Zhao et al. (12) analyzed the serum specific IgE and IgG of nearly 100 children with allergic purpura, and found that food sIgG, food allergen sIgE, inhalant allergens sIgE and tIgE were closely related to the development of the disease in the children, and concluded that avoiding contact with allergens in the clinic could play a therapeutic and preventive role in the disease. In addition, the results showed that the positive rates of food-specific IgG antibodies were higher than those of inhalant allergen IgE, which led some scholars to suggest that “food intolerance-specific IgG antibodies can be a powerful complement to the detection of allergen IgE antibodies.” The rapid onset of IgE-mediated food allergy, with onset of symptoms within minutes to hours and severe anaphylactic shock, is a far cry from the chronic onset of Hashimoto’s thyroiditis, which is not easy to detect.

Compared to IgE-mediated allergies, IgG-mediated food intolerances align more closely with the characteristics of Hashimoto’s thyroiditis. Recent research indicates that food intolerances are not limited to gastrointestinal disorders but are also associated with the development of various diseases, such as psoriasis (13), systemic lupus erythematosus (14), chronic urticaria (15), and many other immune disorders.

The mechanisms underlying food intolerance are diverse and debated. The predominant theory (16, 17) posits that food should be completely digested into low molecular weight nutrients in the gastrointestinal tract. However, intolerance can arise from factors such as the food’s matrix composition, enzyme deficiencies, or imbalances in gut microbiota, which lead to the entry of food as peptides or other large molecules into the intestines. When these large molecules are recognized as foreign by the body, they trigger an autoimmune response. Hu et al. (18) studied seventeen types of wheat to assess whether the digestibility of gluten correlates with its potential allergenicity. Their findings suggest that undigested gluten has significant immunogenicity, which diminishes as the gluten is broken down during digestion, thereby reducing its immune imprint. This research provides indirect evidence supporting the proposed mechanisms of food intolerance. Furthermore, studies by Maleki et al. (19) indicate that the Maillard reaction during peanut processing creates higher molecular weight aggregates, increasing their resistance to digestive breakdown and enhancing their allergenic potential. Consequently, the composition of the food matrix and processing techniques may also play a critical role in sensitization.

All foods have the potential to act as allergens (18, 20), with specific proteins within the foods being the primary culprits. The larger and more complex the protein, the stronger its potential for causing allergies. In this study, eggs and dairy had particularly high rates of food intolerance, with both appearing among the top 14 most intolerant foods out of the 90 tested. The high protein content in eggs and dairy, along with their stable structures that resist heat processing and enzymatic breakdown, may contribute to their high allergenic potential. Additionally, the likelihood of allergic reactions to eggs and dairy appears to diminish with age (8),possibly due to changes in consumption patterns across different life stages.

Daily diet often influences the development of diseases (21–25). Dietary management (26, 27) has been shown to improve nutritional status, quality of life, blood sugar levels, and thyroid function, while effectively reducing the incidence of acute and chronic complications. Many researchers propose the existence of a thyroid-gut axis (28), where different dietary patterns can indirectly or directly influence thyroid function (23, 25) and even be a contributing factor to autoimmune thyroid diseases. A PREDIMED trial by Ruggeri et al. (29), involving a questionnaire survey of 81 HT patients and 119 healthy individuals, revealed that HT patients consumed higher amounts of animal-based foods (especially red meat and processed products), whereas the healthy group consumed more plant-based foods, including legumes.

Modern medical research has recognized that food intolerance is widespread; Kalicanin et al. (30) conducted tests for 125 food-specific IgG antibodies on 74 HT patients and 245 healthy controls. By calculating the food intolerance rates and analyzing the correlation with thyroid function, thyroid-related antibodies, and thyroid volume, they concluded that elevated IgG antibodies have no clinical correlation with the progression of Hashimoto’s thyroiditis to hyperthyroidism or hypothyroidism. Furthermore, the increase in IgG antibodies is not related to the severity of hypothyroidism symptoms; Chen et al. (31) analyzed the correlation between seven thyroid function indicators and the results of 14 food intolerance tests in 45,764 individuals undergoing physical examinations. Their findings indicated that food intolerance is a risk factor for abnormal thyroid function. Eggs, soybeans, crabs, and pork were particularly noteworthy, as intolerance to these four foods correlated with thyroid function abnormalities. Among the study subjects, 24.77% exhibited at least one of the seven thyroid function abnormalities. The highest abnormality rate was observed in TSH levels, followed by TgAb and TPOAb, all of which were statistically significant.

Taken together with previous studies, we consider that food intolerance is associated with thyroid dysfunction in the immune state, rather than being a specific factor that leads to hyperthyroidism or hypothyroidism, so we did not clearly distinguish between combined hyperthyroidism or combined hypothyroidism in our study. Given the high cost of the test, we did not perform it on all patients with Hashimoto’s thyroiditis, but rather on those with severe symptoms, such as high antibody titers, significant changes in thyroid texture, difficult-to-correct thyroid dysfunction, and significant fluctuations in thyroid-stimulating hormone (alternating between hyperthyroidism and hypothyroidism), which may have contributed to the increased positivity rate. The use of a 90-food test in the present study, compared to previous studies that tested 7 or 14 foods, also contributed to the higher overall positive rate of intolerance observed.

This study demonstrates a close correlation between Hashimoto’s thyroiditis and food intolerance. Compared to the healthy population, patients with Hashimoto’s thyroiditis are intolerant to a broader array of foods. Among the 90 foods tested, significant differences in intolerance rates between the two groups were noted for cheddar cheese, soft white cheese, eggs, yogurt, wheat, carrots, pineapple, bananas, cantaloupe, malt, butter, cinnamon, mustard, cow’s milk, crab, goat’s milk, belt fish, grass carp, cucumber, pumpkin, and mushrooms (*P* < 0.05). HT patients showed particularly high intolerance rates to eggs and dairy products, predominantly at levels II and III of intolerance; interestingly, bok choy had a very high intolerance rate (HT group: 71.76%, healthy population: 54.02%), mostly at level I, but the difference between the two groups was not statistically significant (χ^2^ = 6.534, *P* = 0.061), which may be related to dietary habits in the Guangdong region.

Mei et al. (15) employed a treatment regimen combining medication with dietary restrictions (mainly including “alternate diet” “No-Eat” and “Absolute No-Eat”) based on food intolerance test results in 180 patients with chronic urticaria. Their study found that compared to the control group, the quality of life of the treatment group improved, and the duration and frequency of outbreaks decreased. Therefore, future prospective studies could enhance the clinical value of this research. Currently, there are few studies on the correlation between food intolerance and Hashimoto’s thyroiditis, and no observational studies have been conducted in China regarding dietary management changes.

Additionally, the limitations of this study include a small sample size, significant gender imbalance, and a relatively narrow range of tested foods, indicating the need for further in-depth research in the future.
